# Automatic Liver Segmentation from CT Images Using Single-Block Linear Detection

**DOI:** 10.1155/2016/9420148

**Published:** 2016-08-18

**Authors:** Lianfen Huang, Minghui Weng, Haitao Shuai, Yue Huang, Jianjun Sun, Fenglian Gao

**Affiliations:** ^1^Xiamen University, Xiamen, Fujian 361005, China; ^2^Department of Radiology, The 476th Clinic Section, Fuzhou General Hospital of the PLA, Fuzhou, Fujian 350002, China

## Abstract

Automatic liver segmentation not only plays an important role in the analysis of liver disease, but also reduces the cost and humanity's impact in segmentation. In addition, liver segmentation is a very challenging task due to countless anatomical variations and technical difficulties. Many methods have been designed to overcome these challenges, but these methods still need to be improved to obtain the desired segmentation precision. In this paper, a fast algorithm is proposed for liver extraction from CT images with single-block linear detection. The proposed method does not require iteration; thus, the computational time and complexity are decreased enormously. In addition, the initialization is not crucial in the algorithm, so the algorithm's robustness and specificity are improved. The experimental evaluation of the proposed method revealed effective segmentation in normal and abnormal (liver hemangioma and liver cancer) abdominal CT images. The average sensitivity, accuracy, and specificity for liver cancer are 96.59%, 98.65%, and 99.03%, respectively. The results of image segmentation approximate the manual segmentation results by the technical doctor. Moreover, our method shows superior flexibility to newly published method with comparable performance. The advantage of our method is verified with experimental results, which is described in detail.

## 1. Introduction

At present, many postprocessing techniques with information from Computed Tomography (CT) images help confirm diagnoses, biopsies, and morphological anatomies [1–3]. Also these techniques of Magnetic Resonance Imaging (MRI) images are important in the pathophysiology and development of disease [4, 5]. Sharma and Aggarwal [6] discussed the merits and limitations of automated segmentation methods. Postprocessing techniques, with abdominal CT images, are widely used to determine types of liver disease. Liver segmentation from CT images in postprocessing techniques not only is an essential prerequisite, but, by playing an important role in confirming liver function, pathological, and anatomical studies, is also a key technique for diagnosis of liver disease. An accurate liver segmentation method is helpful in diagnosis, treatment, and computer-aided operations with 3D reconstruction techniques. An accurate liver segmentation method is also used to confirm lesion size and degree for specific treatment or the lesion sharp and location for surgical operations, such as radiotherapy.

The level set method, introduced to the field of image processing, image restoration, and target tracking, has been in use since the 1990s. In recent journals [8, 9], the level set method was proposed for liver segmentation with CT images. Also, the level set method was used to segment magnetic resonance images with intensity clustering properties to overcome intensity inhomogeneities in the images [10]. Then, based on energy minimization, the method was improved to estimate joint bias fields and segment magnetic resonance images [11]. To avoid error accumulation, the level set function is usually reinitialized to maintain a Signed Distance Function (SDF) in this method, resulting in greater computational time. In 2010, Li et al. [12] proposed Distance Regularized Level Set Evolution (DRLSE) for image segmentation, which established a SDF near zero level set during evolution, thus simplifying the numerical calculations and saving considerable time. However, in the acquisition process, the details in the CT images were obscured by artifacts, such as the following: strip, motion, beam hardening, ring, and metal artifacts and also the partial volume effect [13], adding to the CT image complexity. Therefore, this method revealed unsatisfactory results in abdominal CT image segmentation.

Assessment of disease attracts many researchers' interest through medical image [15, 16]. Numerous model-based methods, mainly based on local and prior global liver shape knowledge, have been proposed to perform liver segmentation from CT images [17–19]. In 2016, Shi et al. reported a new MLR-SSC prior shape model [14]. Their model achieves better accuracy and superior performance in liver cases having low contrast with neighboring organs and presence of pathologies but requires relatively good initial shapes and significant computational time.

To effectively deal with the above problems, we proposed a single-block linear detection algorithm (SBLDA) for automatic liver segmentation from abdominal CT images. Our proposed method, whose underlying technique is used to segment retinal blood vessels, successfully extracts the liver's edge from CT images [20, 21]. The contribution of our framework may be enumerated as follows:

1. Our algorithm overcomes artifacts and reveals satisfactory segmentation results in abdominal CT images with low contrast.
2. Our method does not require iteration and initialization; thus, computational time decreases and robustness increases.

## 2. Theoretical Formalism

**Figure 1:**
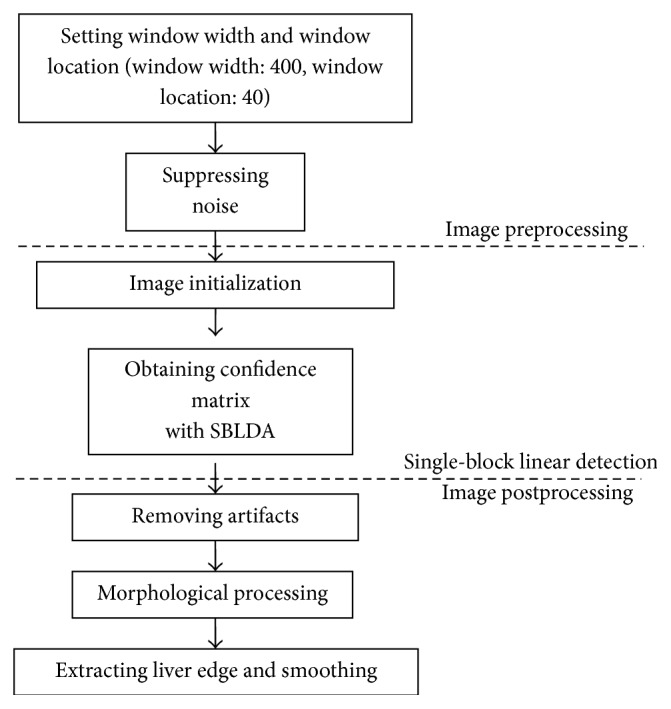
Diagram of the single-block linear detection algorithm.

The diagram of the segmentation method, using single-block linear detection, is shown in Figure 1. The diagram includes three major parts: image preprocessing, liver edge extraction with SBLDA, and image postprocessing. Image preprocessing is subdivided into two steps: setting window width and window location and suppressing noise. During single-block linear detection, we first select the input image as seeds and initialize the confidence matrix with zero and then update the confidence matrix with SBLDA. Image postprocessing includes the following: (1) removing the ring artifacts in abdominal CT images and decreasing misclassifications with morphological processing, (2) reconnecting structures disconnected from the main liver edge with median filtering, and (3) locating the liver area in terms of the liver's anatomic-features.

### 2.1. Suppressing Noise

In image preprocessing, the window width (400) and window location (40) are chosen to better display the original image. The noise in the images may lead to false update of the confidence matrix, which is usually misclassified as liver edge. However, the effect of the noise on segmentation can be reduced by smoothing the original images with median filtering.

**Figure 2:**
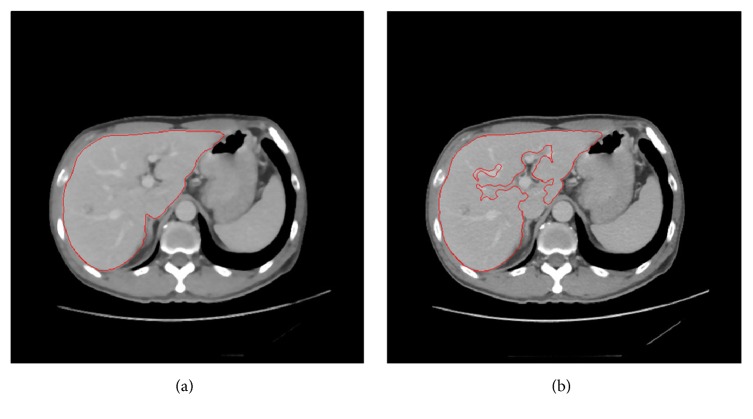
Suppressing noise. The segmentation results with and without performing median filtering (window size: 5 by 5) are shown in (a) and (b), respectively.

Edges are of critical importance to the visual appearance of images, so it is important to preserve the edges while removing noise. The median filtering has been demonstrably to be useful in removing noise but preserving edges for a given fixed window size. Therefore, median filtering is widely used in image preprocessing. The 2D median filtering algorithm is summarized in the form of pseudocode (Pseudocode 1). The segmentation results, with and without median filtering (window size is five by five), are shown in Figures 2(a) and 2(b), respectively. In Figure 2(b), the confidence matrix has a false update, and the noise is misclassified as liver edge.

### 2.2. Seed Initialization in Traditional Algorithm

**Figure 3:**
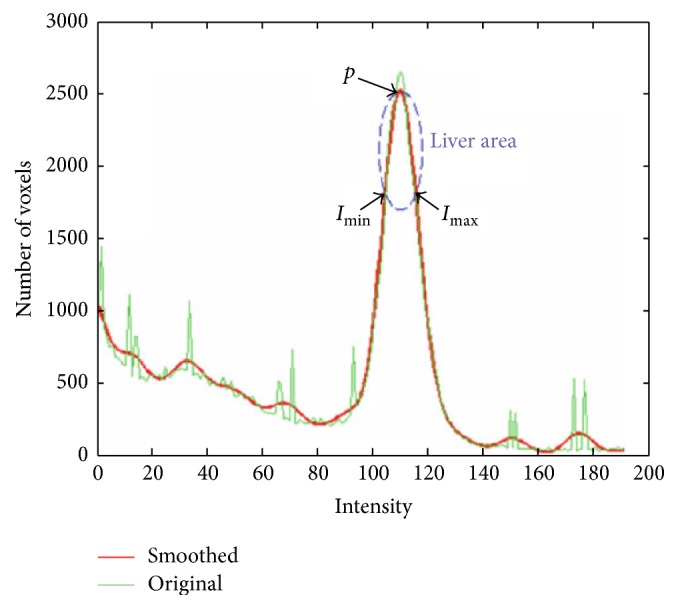
Gray histogram of abdominal liver CT (figure adapted from reference [7]).

In the histogram of the abdominal CT images (Figure 3), assume that *h*(*p*) is the histogram value of the highest pitch and *p* is the corresponding intensity value. The minimal and maximal liver intensity ranges are roughly defined as *p* ± 3 [7]. The intensity of the pixels in the determined range is assigned the value one; the remainders are assigned the value zero. Then, the initialized liver area (seed-image), *V*_*s*_, is created, in which one or more points are usually selected as seeds in some algorithm [22]. Because of the abdominal CT images' complexity and artifacts, a rough liver estimation from CT images with this method is so inefficient that the traditional algorithm yields bad segmentation results. Fortunately, in the SBLDA, it is not crucial or important to determine the liver area before starting the algorithm. We use the original CT image *I* as the seed-image (*V*_s_ = *I*) for SBLDA.

### 2.3. Single-Block Linear Detection Algorithm

During the single-block linear detection process, *w* denotes the detection window size. The pixel CT and block CT values of the input image at position (*x*, *y*) are expressed as *I*(*x*, *y*) and *C*(*x*, *y*), respectively. The block CT value *C*(*x*, *y*) is the average CT values of the corresponding block (block size: *N*), which is defined in (1). Then, ratio parameter of candidate points *R*(*x*, *y*) orientated at *θ* degree (*θ* = 0°, 45°, 90°, 135°, 180°, 225°, 270°, 315°), the maximum of which is stored in *R*_*m*_, is approximated using (2). When *R*_*m*_ exceeds the predefined threshold, *T*, the corresponding confidence matrix *C*_*w*_(*x*, *y*) is set to one; otherwise, the matrix is set to zero (see (4)):

(1) Cx,y=1N−12∑i=0N−1 ∑j=0N−1Ix+i,y+j

(2) Rx,y=Cx±1,y+w+Cx±1,y−w−2Ix±1,y2Ix±1,yCx+w,y±1+Cx−w,y±1−2Ix,y±12Ix,y±1Cx±1+w,y±1−w+Cx±1−w,y±1+w−2Ix±1,y±12Ix±1,y±1Cx±1+w,y∓1+w+Cx±1−w,y∓1−w−2Ix±1,y∓12Ix±1,y∓1

(3) Rm=max⁡Rx,y

(4) Cwx,y=1,Rm≥T0,Rm<T.

**Figure 4:**
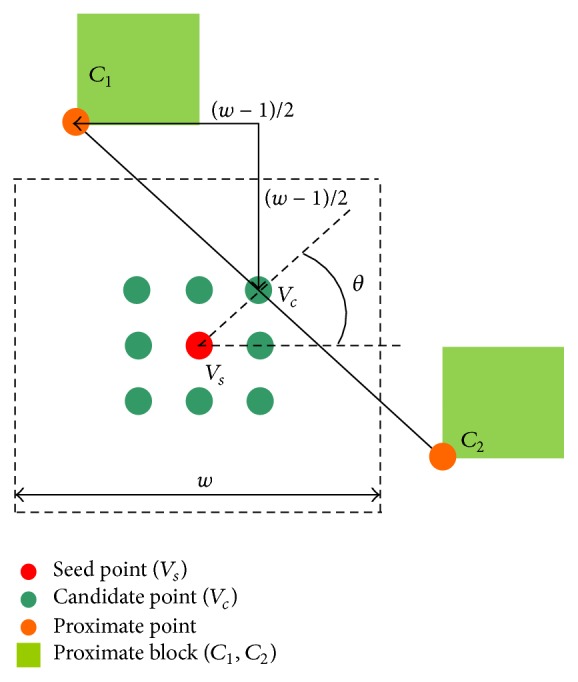
The estimation of ratio parameters.

The estimation of the ratio parameter is shown in Figure 4. *V*_*s*_ is the CT value of any selected point in SBLDA. The value of confidence matrix for this selected point is initialized to zero (*C*_*w*_ = 0). *V*_*c*_ is the CT value of the candidate point orientated at *θ* degree. An example of *θ* = 45° is given in Figure 4. *C*_1_ and *C*_2_ are the block CT values of the points located *w*/2 size away from *V*_*c*_ in opposite directions (*w* = 7, *N* = 3); the ratio parameters for this point, *R*_*c*_ (45 degrees, e.g.), are expressed as *R*_*c*_ = (*C*_1_ + *C*_2_ − 2*V*_*c*_)/2*V*_*c*_ (defined in (2)). When the candidate point belongs to boundary points, the amplitude value of *R*_*c*_ is large. If the maximum value of |*R*_*c*_|, orientated at eight directions, is greater than threshold *T*, the corresponding value of confidence matrix for *V*_*s*_ is updated to one (*C*_*w*_ = 1).

**Figure 5:**
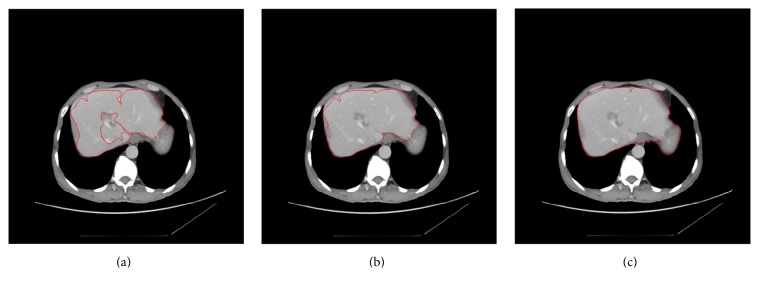
The segmentation results of a CT image with various values of* T* (*T* = 0.8, 0.9, and 1.2) are shown in (a), (b), and (c), respectively.

The segmentation results of a CT image, with various values of *T* (*T* = 0.8, 0.9, and 1.2), are shown in Figure 5. The value of threshold *T* plays a key role in SBLDA. If the value of *T* is too small, the liver area with noise would be misclassified as liver edge (Figure 5(a)). If the value of *T* is too large, some liver edges would be missed and then nonliver area would be misclassified as liver (Figure 5(c)). The result, with optimal *T*, is shown in Figure 5(b). The appropriate value for the threshold *T* is described in Section 2.

In this paper, the ratio parameter is well evaluated if a point is at the boundary in the proposed algorithm; thus the algorithm is iteration-free. In addition, this algorithm's segmentation accuracy is greatly improved, especially for weak edge areas.

### 2.4. Removing Artifacts

**Figure 6:**
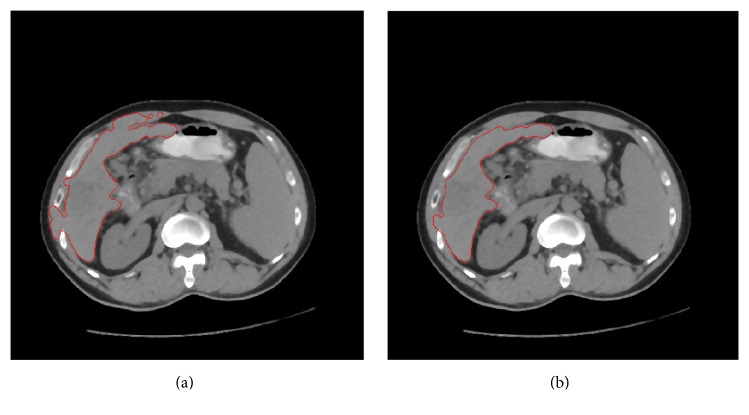
Artifacts removal. The segmentation results without ring artifacts removal and with ring artifacts removal were shown in (a) and (b), respectively.

Because of random disturbance, there are many artifacts in CT images, such as ring and strip artifacts. Not only are ring artifacts and the liver closely linked, but they also have very similar intensity. In some cases, ring artifacts usually affect segmentation results. To obtain the mask of the edge, M, we performed a morphological open operation on the edge of the original CT image. Then, by the product of M and the original image, ring artifacts in the CT images were removed. The segmentation results, with and without performing morphological filtering, are shown in Figure 6.

### 2.5. Morphological Processing and Liver Segmentation

**Figure 7:**
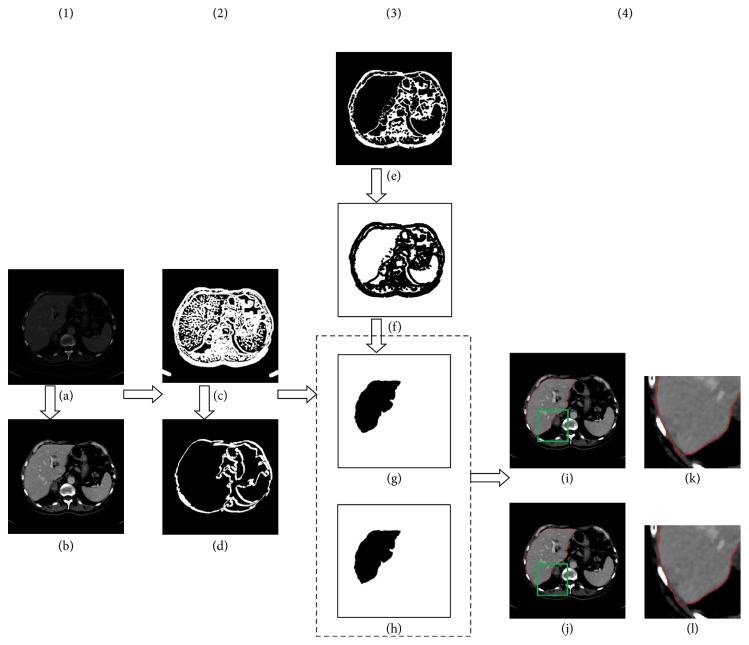
An example of SBLDA. The subparts of image preprocessing, segmentation with SBLDA, image postprocessing, and final result were indicated by (1)–(4). The regions (green rectangles) were zoomed in to validate the importance of removing artifacts.

Based on the liver's anatomical features, the liver is in the left region of the abdominal CT image and has the largest area. The liver edge is obtained after morphological processing based on the confidence matrix calculated by SBLDA. An example of the segmentation with SBLDA is shown in Figure 7. The CT image used in this example is plane 64 of database 19 from publicly available database (3Dircadb1). The original input and output images with preprocessing are shown in Figures 7(a) and 7(b), respectively. In Figure 7(c), the confidence matrix is displayed after SBLDA. At the same time, the mask M (Figure 7(d)), used to remove ring artifacts and improve the segmentation accuracy, is extracted. The confidence matrix is performed with erode operation to remove the error edge points and then retain the maximum connected region (Figure 7(e)). The matrix shown in Figure 7(e) is inverted and eroded, and the new matrix is shown in Figure 7(f). Then, by retaining the maximum connected region and smoothing with a Gaussian filter, the liver edge is extracted with and without mask, M, as shown in Figures 7(g) and 7(h), respectively. The corresponding results are shown in Figures 7(i) and 7(j). The green rectangles are enlarged and shown on the right.

## 3. Experimental Results

The experimental environment of our system was as follows: the CPU was an Intel(R) Core(TM) Quad Q8400; the basic frequency was 2.66 GHz; the hard disk was 300 GB; the RAM was 6 GB; the main operating system was Windows 7. This study included abdominal CT images (4 normal, 4 cancer, and 4 hemangioma liver samples) acquired on a LightSpeed Volume Computed Tomography (VCT) GE healthcare CT scanner (with halo detector, 64 slices, General Electric Medical System). Each image was acquired at 512 × 512 pixels. We used a set of manual segmentations for comparison with the segmentations generated by our proposed method. To evaluate our method's performance, we used a publicly available database (3Dircadb1) that includes 20 CT scans (10 women and 10 men) with corresponding manual segmentation results provided by the French Research Institute against Digestive Cancer (IRCAD). This database was composed of 3D CT scans, in which 75% of the cases include hepatic tumors. Each matrix size in a plane was 512 × 512 pixels. Experimental results validated the feasibility and specificity of SBLDA for liver segmentation.

**Figure 8:**
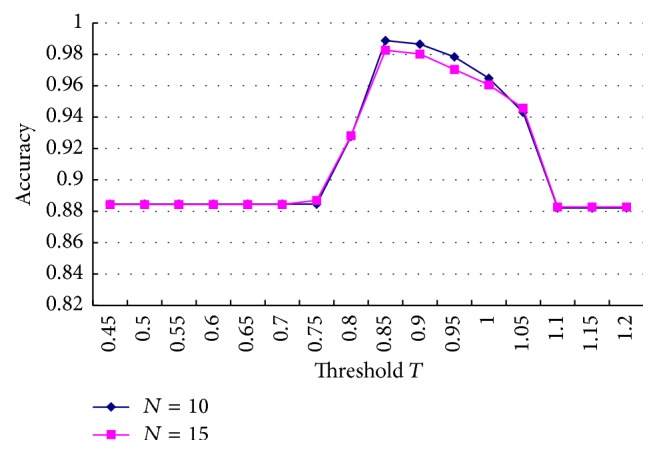
The average accuracy of segmentation with various values of *T*.

In Figure 8, the average accuracy of segmentation was estimated for various values of *T* with *N* random CT images (*N* = 10, 15). To have a meaningful estimation, CT liver images with and without tumor (1 : 1) were selected. Both curves in Figure 8 demonstrated that the accuracy was higher than 96% when threshold *T* was between 0.82 and 1. During liver segmentation, an eight-pixel neighborhood was used to compute the ratio parameter with the best values (*T* = 0.85, *w* = 9) in all cases. Outliers were further removed with morphological filtering using a disk structuring element with a radius of two pixels. To remove ring artifact, morphological filtering with a disk having a radius of 20 pixels was used. The disconnected structures of the main liver edge were reconnected with median filtering.

**Figure 9:**
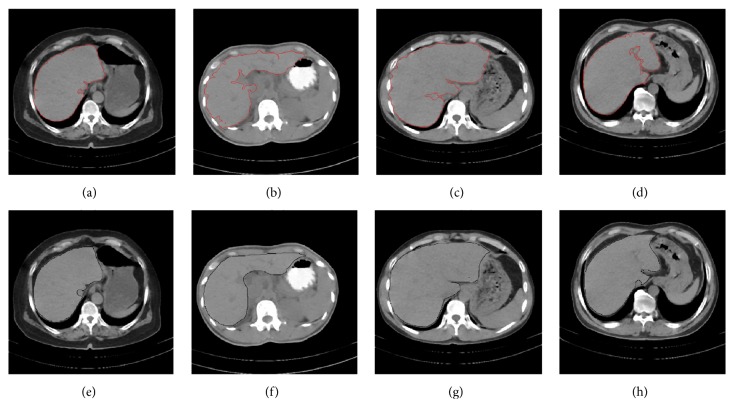
Segmentation results of normal CT images. (a–d) The segmentation results with proposed method. The liver areas marked by technical doctor are shown in (e–h).

**Figure 10:**
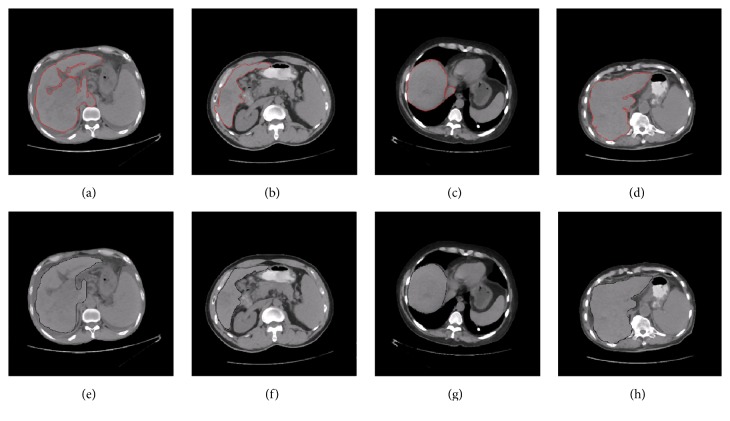
Segmentation results of CT images with liver cancer. The segmentation results with SBLDA and doctor are shown in (a–d) and (e–h), respectively.

**Figure 11:**
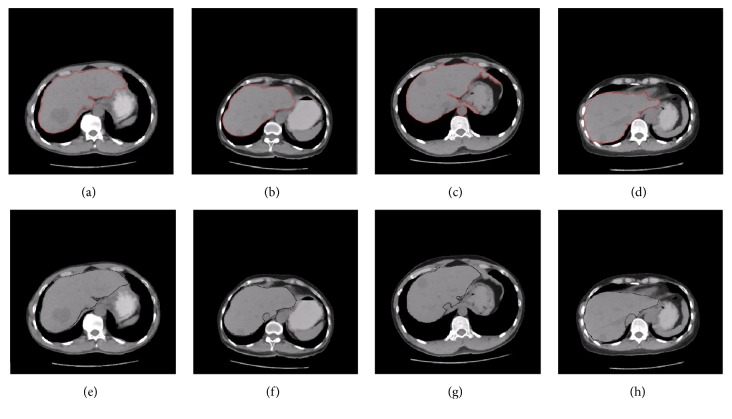
Segmentation results of CT images with liver hemangioma. The segmentation results with SBLDA and doctor are shown in (a–d) and (e–h), respectively.

**Figure 12:**
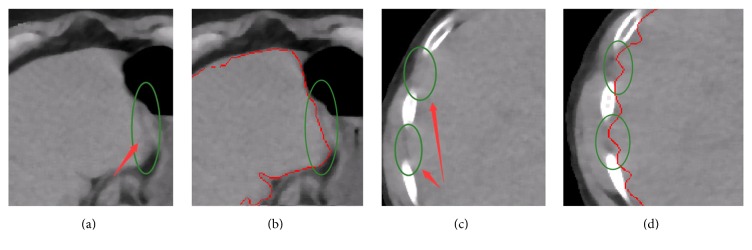
Weak edge segmentation results of normal liver tissue samples. (a) and (c) mark the neglected weak boundary (green oval). (b) and (d) show the segmentation results of weak boundary with the reported algorithm.

**Figure 13:**
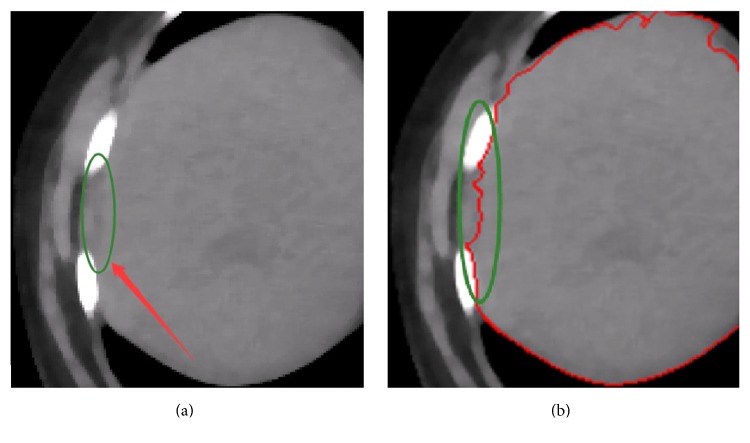
Weak boundary segmentation results of liver cancer samples. (a) marks the neglected weak boundary (green oval). (b) shows the weak boundary segmentation results with the proposed algorithm.

Figures 9(a)–9(d), 10(a)–10(d), and 11(a)–11(d) show the segmentation results of the normal and abnormal (liver cancer and liver hemangioma) abdominal CT images with SBLDA. For comparison, the manual segmentation results were shown at the bottom of the corresponding figures. The weak edge in the CT image in Figure 9(a), which was difficult for an observer to classify, was enlarged and indicated by red solid arrows in Figure 12(a). The satisfactory result of weak edge segmentation using our proposed method was shown in Figure 12(b) (green solid oval). Figures 12(c) and 12(d) show the weak edge and the corresponding segmentation result of the CT image shown in Figure 9(c). The weak edge of the sample with liver cancer (Figure 10(c)) was enlarged and shown in Figure 13(a); the segmentation results were shown in Figure 13(b). The experimental results in Figures 12 and 13 show the efficiency of our method for weak edge subtraction.

In the liver segmentation process, true positives (TP) and true negatives (TN) were correctly classified. A false positive (FP) indicates that a nonliver pixel is classified as a liver pixel. A false negative (FN), in which a liver pixel is classified as a nonliver pixel, is also an error. In this study, we used three statistical measures of the performance of a binary classification to evaluate our proposed algorithm: sensitivity, specificity, and accuracy. Sensitivity measures the proportion of true positives, whereas specificity measures true negatives. Accuracy measures the proportion of correctly segmented pixels to the total number of pixels in a CT image. Let *P* and *N* represent the total number of pixels in the liver and nonliver; then sensitivity, specificity, and accuracy are defined as follows: sensitivity = TP/(TP + FN), specificity = TN/(TN + FP), and accuracy = (TP + TN)/(*P* + *N*).  Table 1 presents the quantitative validation from normal and abnormal CT image segmentation. The advantage of SBLDA was evident in terms of sensitivity, specificity, and accuracy.

To evaluate the accuracy and performance on the 3Dircadb1 database, we compared our method with Shi's method (MLR-SSC). The following five parameters were selected as the basis for the quantitative analysis: (1) volumetric overlap error (VOE), (2) signed relative volume difference (SRVD), (3) average symmetric surface distance (ASD), (4) root mean square symmetric surface distance (RMSD), and (5) maximum symmetric surface distance (MSD). These parameters were represented as mean and standard deviation of the overall datasets. An open source code, provided by the “MICCAI 2007 Grand Challenge” workshop, was used to calculate the quantitative comparative results. The average computation time in the training and testing stages was selected to evaluate the computational complexity. Before the segmentation of each dataset, the optimal value of *T* was estimated by 10 random CT images in the dataset. Quantitative comparative results, where N/A stood for no spending time, were shown in Table 2. Our method consumed less time to obtain higher performance in the VOE (7.84 versus 8.74). The value of other four parameters was close to those reported by MLR-SSC algorithm. However, the consumption of time was a significant reduction.

Our method's average computation time, in the running stage, is 36.4 min per database. Shi's method, however, requires much time (about 400 h) in the training stage and 8.5 min in the testing stage [14]. Because there is no offline training stage in our method, the computational time and complexity are decreased enormously. In MLR-SSC, the initial shapes are relatively poor in some cases with large liver tumors, leading to significant segmentation error. However, in our method, the segmentation result is not affected by initialization.

**Figure 14:**
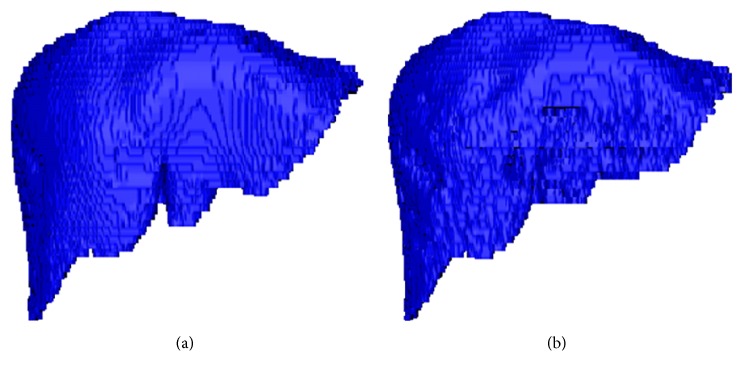
3D visual liver of referential segmentation results (a) and segmentation results with our proposed (b).

Shown in Figure 14 was a 3D visual example of segmentation results, based on datasets 19 from 3Dircadb1 database. The referential segmentation results of a 3D visual liver (included in the 3Dircadb1 database) are shown in Figure 14(a). Promising segmentation results, obtained with our proposed method, are shown in Figure 14(b).

## 4. Conclusion

In this paper, we proposed a CT liver segmentation method, based on a single-block linear detection algorithm, and verified the method's feasibility with theoretical and experimental results. Because the algorithm starts without predefined seeds' images, initialization does not affect its performance. Moreover, this method uses a noniteration process, thereby saving total CPU running time. Advantages of the method are revealed in the method's easily neglected weak edge. In addition, the method generates a higher average specificity (98.65%) and accuracy (99.03%) for liver edge extraction from CT abdominal images (liver cancer). The results of image segmentation approximate a technical doctor's manual segmentation results. Quantitative results are as similar as those for MLR-SSC. Compared with MLR-SSC, our method not only saves considerable runtime but also is not affected by initial shapes.

Despite the available results, our proposed method could be improved further in the following ways: (1) much segmentation error exists in CT images with relatively small liver regions; (2) when the tumors are at the edge of the liver, tumor extraction might fail. That, extraction of only liver without tumor would be helpful to only segment healthy organs, such as transplantation candidates. Future work will focus on overcoming the above challenging situation.

## Figures and Tables

**Pseudocode 1:**
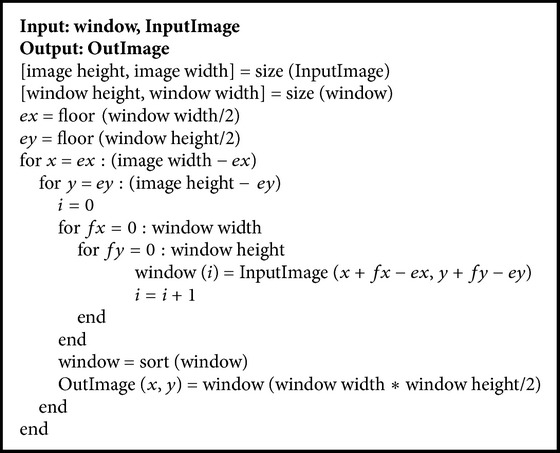
Pseudocode of median filtering algorithm.

**Table 1:** Sensitivity, specificity, accuracy, and the corresponding average of the proposed method.

	Normal				
	1	2	3	4	Average
Sensitivity	0.9939	0.9927	0.9695	0.9894	**0.9864 **
Specificity	0.9947	0.9815	0.9843	0.9924	**0.9882 **
Accuracy	0.9946	0.9837	0.9824	0.9920	**0.9882 **

	*Liver cancer*				
	1	2	3	4	Average

Sensitivity	0.9674	0.9732	0.9727	0.9504	**0.9659 **
Specificity	0.9912	0.995	0.9874	0.996	**0.9865 **
Accuracy	0.9902	0.9937	0.9858	0.9916	**0.9903 **

	*Liver hemangioma*				
	1	2	3	4	Average

Sensitivity	0.9782	0.9827	0.991	0.9827	**0.9837 **
Specificity	0.9844	0.981	0.995	0.9893	**0.9874 **
Accuracy	0.9838	0.9811	0.9947	0.9886	**0.9870 **

**Table 2:** Quantitative results between our method and newly proposed methods based on the 3Dircadb1 database.

Method	VOE (%)	SRVD (%)	ASD (mm)	RMSD (mm)	MSD (mm)	Training time (hour)	Testing time (min)	CPU (GHz)
Shi et al. [14]	8.74 ± 2.37	2.41 ± 1.71	1.45 ± 0.37	2.55 ± 0.59	26.91 ± 7.72	400	8.5	2.33
Our method	7.84 ± 2.95	3.42 ± 2.11	1.97 ± 1.02	4.71 ± 1.96	37.05 ± 9.82	N/A	36.4	2.66

## References

[1] Li X., Wang X., Dai Y., Zhang P. (2015). Supervised recursive segmentation of volumetric CT images for 3D reconstruction of lung and vessel tree. *Computer Methods and Programs in Biomedicine*.

[2] Selver M. A. (2014). Segmentation of abdominal organs from CT using a multi-level, hierarchical neural network strategy. *Computer Methods and Programs in Biomedicine*.

[3] Mala K., Sadasivam V., Alagappan S. (2015). Neural network based texture analysis of CT images for fatty and cirrhosis liver classification. *Applied Soft Computing C*.

[4] Wu C., Honarmand A. R., Schnell S. (2016). Age-related changes of normal cerebral and cardiac blood flow in children and adults aged 7 months to 61 years. *Journal of the American Heart Association*.

[5] Wu C., Ansari S. A., Honarmand A. R. (2015). Evaluation of 4D vascular flow and tissue perfusion in cerebral arteriovenous malformations: influence of Spetzler-Martin grade, clinical presentation, and AVM risk factors. *American Journal of Neuroradiology*.

[6] Sharma N., Aggarwal L. M. (2010). Automated medical image segmentation techniques. *Journal of Medical Physics*.

[7] Kumar S. S., Moni R. S., Rajeesh J. (2013). Automatic liver and lesion segmentation: a primary step in diagnosis of liver diseases. *Signal, Image and Video Processing*.

[8] Wang J. K., Sun J. J., Gao F. L. (2014). Automatic fatty liver diagnostic system in CT image based on DRLSE method. *Journal of Xiamen University*.

[9] Gao F. L., Huang L. F., Wang J. K., Shuai H. T., Sun J. J., Huang Y. (2014). Automatic liver lesion extraction from CT images based on distance regularized level set evolution and region growing. *Applied Mechanics and Materials*.

[10] Li C., Huang R., Ding Z., Gatenby J., Metaxas D. N., Gore J. C. (2011). A level set method for image segmentation in the presence of intensity inhomogeneities with application to MRI. *IEEE Transactions on Image Processing*.

[11] Li C., Gore J. C., Davatzikos C. (2014). Multiplicative intrinsic component optimization (MICO) for MRI bias field estimation and tissue segmentation. *Magnetic Resonance Imaging*.

[12] Li C., Xu C., Gui C., Fox M. D. (2010). Distance regularized level set evolution and its application to image segmentation. *IEEE Transactions on Image Processing*.

[13] Jiang G. P., Qin W. J., Zhou S. J., Wang C. M. (2015). State-of-the-art in medical image segmentation. *Chinese Journal of Computers*.

[14] Shi C. F., Cheng Y. Z., Liu F., Wang Y. D., Bai J., Tamura S. (2016). A hierarchical local region-based sparse shape composition for liver segmentation in CT scans. *Pattern Recognition*.

[15] Wu C., Schnell S., Markl M., Ansari S. A. (2015). Combined DSA and 4D flow demonstrate overt alterations of vascular geometry and hemodynamics in an unusually complex cerebral AVM. *Clinical Neuroradiology*.

[16] Wu C., Schoeneman S. E., Kuhn R. (2015). Complex alterations of intracranial 4-dimensional hemodynamics in vein of galen aneurysmal malformations during staged endovascular embolization. *Operative Neurosurgery*.

[17] Afifi A., Nakaguchi T. (2012). Liver segmentation approach using graph cuts and iteratively estimated shape and intensity constrains. *Medical Image Computing and Computer-Assisted Intervention—MICCAI 2012*.

[18] Wang J., Cheng Y., Guo C., Wang Y., Tamura S. (2016). Shape-intensity prior level set combining probabilistic atlas and probability map constrains for automatic liver segmentation from abdominal CT images. *International Journal of Computer Assisted Radiology and Surgery*.

[19] Eapen M., Korah R., Geetha G. (2016). Computerized liver segmentation from CT images using probabilistic level set approach. *Arabian Journal for Science and Engineering*.

[20] Nguyen U. T. V., Bhuiyan A., Park L. A. F., Ramamohanarao K. (2013). An effective retinal blood vessel segmentation method using multi-scale line detection. *Pattern Recognition*.

[21] Vlachos M., Dermatas E. (2010). Multi-scale retinal vessel segmentation using line tracking. *Computerized Medical Imaging and Graphics*.

[22] Li X., Luo S., Li J. (2013). Liver segmentation from CT image using fuzzy clustering and level set. *Journal of Signal and Information Processing*.

